# The geometry of structural equilibrium

**DOI:** 10.1098/rsos.160759

**Published:** 2017-03-22

**Authors:** Allan McRobie

**Affiliations:** Cambridge University Engineering Department, Trumpington Street, Cambridge CB2 1PZ, UK

**Keywords:** Maxwell reciprocal diagrams, Rankine reciprocal diagrams, graphic statics, three-dimensional frames, Clifford algebra

## Abstract

Building on a long tradition from Maxwell, Rankine, Klein and others, this paper puts forward a geometrical description of structural equilibrium which contains a procedure for the graphic analysis of stress resultants within general three-dimensional frames. The method is a natural generalization of Rankine’s reciprocal diagrams for three-dimensional trusses. The vertices and edges of dual abstract 4-polytopes are embedded within dual four-dimensional vector spaces, wherein the oriented area of generalized polygons give all six components (axial and shear forces with torsion and bending moments) of the stress resultants. The relevant quantities may be readily calculated using four-dimensional Clifford algebra. As well as giving access to frame analysis and design, the description resolves a number of long-standing problems with the incompleteness of Rankine’s description of three-dimensional trusses. Examples are given of how the procedure may be applied to structures of engineering interest, including an outline of a two-stage procedure for addressing the equilibrium of loaded gridshell rooves.

## 1. Introduction

Over the past half century, the field of structural analysis has become increasingly a matter of matrix-based linear algebra computation. However, by generalizing methods developed over a century ago by Maxwell, Rankine, Klein and others, this paper provides an alternative approach based almost purely on geometry. A theory of graphic statics is presented which is applicable to the static equilibrium of rather general three-dimensional frames. We begin with some comparatively abstract mathematics to give a formal statement of the general theory, before giving examples to demonstrate how it is practical and applicable to real structures. Although the theory will use algebra, the final construction will be diagrammatic, giving geometric visualization of the various objects such as forces and moments.

The overall setting will be four-dimensional Clifford algebra. That this is also known as geometric algebra (or GA) accords with the view that this is the appropriate language in which to construct a purely geometric theory. Perhaps remarkably, the description of structural equilibrium that emerges bears marked similarities to the geometric algebra description of electromagnetism.

## 2. Background

### 2.1. Graphic statics

The origin of graphic statics can be traced at least as far back as 1725, when Varignon [1] observed that the equilibrium of a simple two-dimensional truss requires the bar force vectors meeting at each node to form a closed polygon, and that these force polygons could be assembled into a larger figure known as the *force diagram*. The next advances came in the late nineteenth and early twentieth century with the work of Maxwell [2,3], Rankine [4,5], Culmann [6], Cremona [7], Klein [8] and many others. A good description of the chronology is provided by Kurrer [9].

Maxwell’s 1864 and 1870 papers on reciprocal diagrams are particularly important. There are two reciprocal diagrams, the *form* diagram and the *force* diagram. The form diagram shows the geometry of the two-dimensional truss and the force diagram is the assemblage of nodal force polygons for an equilibrium state of stress. As recently described by Mitchell *et al.* [10] and McRobie *et al.* [11], Maxwell observed that the nodal force polygons could be assembled to make a force diagram *if and only if* the form diagram was the two-dimensional projection of a three-dimensional polyhedron, in which case the polyhedron may be interpreted as a piecewise linear Airy stress function.

Rankine [5] generalized Maxwell’s construction for two-dimensional trusses to the case of three-dimensional trusses. (By a truss we mean a structure with pin-jointed connections whose members carry only axial forces. Later we shall use the term ‘frame’ to mean a structure whose joints may transmit moments and whose members may carry a combination of axial and shear forces and torsional and bending moments. Note that this modern terminology differs from Maxwell’s and Rankine’s usage wherein pin-jointed trusses were referred to as frames.)

In Maxwell’s construction, the force in a bar is given by the *length of a line* in the two-dimensional reciprocal force diagram, and that line is *perpendicular* to the original bar. In Rankine’s construction, the force in a bar is given by the area of a polygon in the three-dimensional reciprocal force diagram, and that polygon is *perpendicular* to the original bar.

It should be mentioned that Cremona [7] used an alternative convention. For two-dimensional trusses, a Cremona force diagram is simply a 90^°^ rotation of the Maxwell force diagram, such that forces are now *parallel* to their corresponding bars. For three-dimensional trusses, Cremona also represents forces as lines parallel to their corresponding bars, and thus a Cremona three-dimensional force diagram is a fundamentally different object than a Rankine three-dimensional force diagram. In this paper, we follow the perpendicular convention of Maxwell’s lines and Rankine’s polygons and generalize these to the case of three-dimensional frames.

The generalization of Maxwell’s reciprocal diagrams to the case of two-dimensional frames has been presented by Williams & McRobie [12], which showed how bending moments in two-dimensional frames could be represented by a discontinuous Airy stress function. Early attempts to generalize this to three-dimensional frames were presented by McRobie & Williams [13], which looked at the discontinuous limit of the 1870 Maxwell–Rankine stress function (this being one of the two three-dimensional stress functions defined in Maxwell [3], and specifically the one which corresponds to the Rankine three-dimensional construction). McRobie & Williams [14] abandoned the continuous-to-discontinuous approach, and simply defined, *ab initio*, its own ‘discontinuous’ stress function applicable to three-dimensional frames. The paper here puts that final approach on much firmer theoretical foundations. The theoretical framework here is inherently four dimensions, and is most readily described using Clifford (or Geometric) algebra [15]. This new perspective suggests that the stress function ‘discontinuities’ are merely an artefact of projecting a continuous four-dimensional object down to three dimensions.

It is hoped that the present paper is a significant contribution to the progress that has been made across the field of graphic statics in more recent years. This began with the deeply mathematical work on structural topology at Toronto from the 1970s of Baracs, Crapo and Whiteley (e.g. [16]; H. Crapo & W. Whiteley 1994, unpublished draft) which developed into structural rigidity theory. More recently, the subject has received renewed interest as a design tool, the ability to visualize forces being of particular appeal to architects. The standard text is Allen & Zalewski [17], but with much progress currently emerging from groups around Ochsendorf at MIT and Block at ETH [18,19] where applications are typically to the design of structural masonry. Mazurek *et al.* [20] and Beghini *et al.* [21] are examples of how the methods may be applied to structural optimization problems. Other recent work includes that of Micheletti [22], Angelillo *et al.* [23], Fraternali *et al.* [24,25] and Akbarzadeh [26–28], with the recent special issue of the *International Journal of Space Structures* [29] containing many more examples.

### 2.2. Clifford algebra

**Figure 1.**
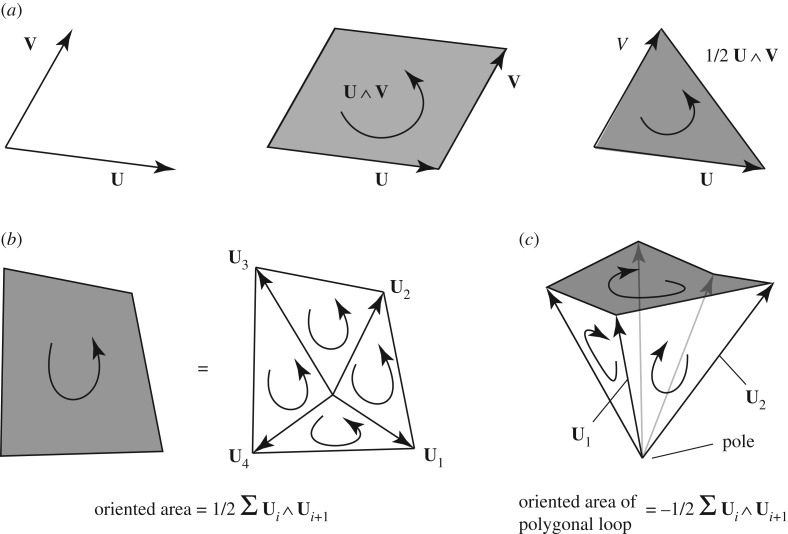
(*a*) The wedge product of vectors **U** and **V** creates the bivector **U**∧**V**, the oriented area defined by sweeping **V** along **U**. (*b*) The oriented area of a plane polygon may be determined by triangulating it, and taking the sum of the oriented areas of the triangles, all orientations having the same sense. (*c*) The oriented area of a general (possibly non-planar or ‘gauche’) polygonal loop may be determined by coning the loop, and then taking (the negative of) the sum of the oriented areas of the triangular sides of the pyramidal ‘cone’.

Clifford algebra (or GA) is an alternative mathematical framework for describing geometry than the vector algebra of Gibbs and Heaviside that is more familiar to most engineers. Clifford algebra shares many features (such as dot products) with vector analysis, but it also possesses additional objects, such as bivectors, trivectors and more general multivectors together with additional operations such as the Clifford product and the wedge product. However, almost the only non-Gibbs item required here is that of a *bivector* created by the wedge product **U**∧**V** of two vectors **U** and **V**. This is the oriented area created when one vector **V** is swept along the other, **U** (figure 1*a*). While similar to a three-dimensional vector cross product, this applies in a space of any dimension. In three dimensions, the bivector **U**∧**V** is the oriented area itself, while **U**×**V** is the *vector* normal to, and of magnitude equal to, that area.

Since the wedge product is also the multiplication operation of the exterior or Grassmann algebra of a vector space, it could be argued that the geometric framework developed in this paper is not a Clifford algebra description, but one that simply employs exterior algebra. However, since the description here is contained within Clifford algebra, we retain the Clifford nomenclature. Moreover, the additional machinery of Clifford algebra may prove to be useful in later developments.

The usefulness of bivectors and wedge products for our current work is how they allow immediate generalization to three-dimensional frames of Rankine’s theory for three-dimensional trusses. In a Rankine diagram for a three-dimensional truss, the axial force in a member is represented by the area of a reciprocal plane polygon orthogonal to the original bar cross-section. In the theory presented here, we employ far more general polygons to represent stress resultants. Using the bivector description, the oriented area of any polygon is readily defined. We begin with a plane polygon, such as the one shown in figure 1*b*. Let the polygon nodes be defined by the vectors {**U**_*i*_} labelled cyclically. Trivially, the oriented area of the polygon is the sum of the oriented areas of the constituent triangles, all of which have the same orientation as the oriented area of the original polygon. Since the oriented areas of the triangles are equal to half the wedge product of the nodal vectors, taken pairwise cyclically, we obtain that the oriented area of the polygon is

2.1 $$\text{Oriented area of polygon}=\frac{1}{2}\sum {\text{U}}_{i}\wedge {\text{U}}_{i+1}.$$

These concepts readily generalize to a polygonal loop in a space of any dimension. Furthermore, the polygon may be what Maxwell called ‘gauche’—i.e. the nodes of the polygonal loop need not lie in a single plane. To obtain the oriented area of a polygonal loop, we first ‘cone’ the loop. That is, we construct vectors from an arbitrary origin (the ‘pole’) to the nodes of the polygonal loop. This creates a polyhedron resembling some form of abstract pyramidal gemstone, with the possibly-gauche polygon as the pyramidal base, surrounded by a garland of triangular facets connecting to the pole (figure 1*c*).

In a space of any dimension, the sum of the oriented areas of the faces of any closed polyhedron is necessarily zero. This is a geometric statement of a familiar notion in three-dimensional hydrostatic equilibrium: any closed object is in equilibrium under the action of a uniform pressure that acts normal to its surface. It trivially follows that the oriented area spanned by the polygonal loop is given by the negative of the sum of the oriented areas of the triangular sides.

2.2 $$\mathrm{Oriented}\;\mathrm{area}\;\mathrm{spanned}\;\mathrm{by}\;\mathrm{polygonal}\;\mathrm{loop}=-\frac{1}{2}\sum {\text{U}}_{i}\wedge {\text{U}}_{i+1}.$$

The negative sign arises because the orientation of a face is indicated by the direction of arrows that circumnavigate the loop of lines that form its boundary. Any polyhedral edge belongs to two and only two face boundaries, and the orientation of the faces are such that the arrows cancel along each edge.

If an edge of the polygonal loop is a curved line, then the above formula may still be applied by approximating the curve by many short straight line segments, and taking the limit as the segment lengths shrink.

Note also that the oriented area is defined by the loop of one-dimensional lines around the polygon edge, independent of whatever two-dimensional surface is chosen to span the loop. That is, the polygonal loops of lines become the fundamental objects of interest. Although we shall use the nomenclature of ‘faces’ throughout this paper, and we may choose to imagine some bubble-like two-dimensional surface spanning each loop, strictly, by a ‘face’ we will mean a polygonal loop.

With respect to structural analysis, the advantage of this Clifford algebra description is that in four dimensions, there are *six* independent bivectors: areas in three of the bivector components can represent the general force (one axial and two shear components) at a bar cross section, with the other three bivector components representing the general moment (one torsion and two bending components) at that cross section. The geometric picture, then, is that the oriented areas of various geometric objects we define will correspond to a set of stress resultants in static equilibrium, and the Clifford algebra provides a natural method for computing the values of the various quantities.

For further background on the use of Clifford algebra in physics generally, the reader is referred to Hestenes [15], and a starting point for its application to structural mechanics is McRobie & Lasenby [30], which translated Simo and Vu-Quoc’s Lie algebra description [31,32] of rod dynamics into the language of bivectors. There, though, the setting was three-dimensional Clifford algebra: forces were represented by vectors and moments by bivectors, with the fourth dimension of time being treated by integration of the resulting ordinary differential equations. In the current paper, the concerns are purely of static equilibrium, and the ‘fourth dimension’ is required to represent the stress function.

## 3. The new description

We now proceed towards the formal statement of the underlying geometric framework of this new description of structural equilibrium. Key to the construction is the definition of two topologically dual 4-polytopes which are then combined to create a larger 4-polytope which will be called the Corsican sum. One polytope *P*_*X*_ represents the structure, and may be thought of as the ‘form diagram’. The other polytope *P*^*A*^ plays a role somewhat analogous to the ‘force diagram’ of traditional Rankine three-dimensional graphic statics (although, more strictly, here the polytope *P*^*A*^ defines a stress function). The Corsican sum of *P*_*X*_ and *P*^*A*^ is a four-dimensional generalization of the three-dimensional Rankine–Minkowski sum that was introduced by McRobie [33], such a sum being a single object which contains both the ‘form’ and the ‘force’ information.

### 3.1. Definitions

The first term requiring explanation is 4-polytope. Loosely, this is the four-dimensional generalization of the familiar notion a three-dimensional polyhedron. However, the usage here is very general, and we define it from bottom up, starting with nodes.

A *node* *J* is simply a point in the four-dimensional space, which may be denoted by a coordinate vector **X**_*J*_ or **A**^*J*^ for *P*_*X*_ and *P*^*A*^, respectively.

A *bar* *IJ* is a line connecting the two nodes *I* and *J*. It need not be a straight line but may curve in any direction. A bar, then, is a segment of a space curve through four-dimensional space.

By a *face* *IJK*…*N*, we mean any surface spanning a polygonal loop of the bars *IJ*,*JK*,…*MN*,*NI*. Since bars may be curved, the polygon may have curvilinear edges, and in particular, the polygon may be what Maxwell called a *gauche* polygon, meaning that it may not lie within a single plane. It will typically not be necessary to define the exact form of this surface: it will usually be sufficient merely to define the loop of bars that define the polygonal edge. A *face*, then, is a surface spanning a one-dimensional loop in a four-dimensional space, but it is the polygonal loop that is of greatest importance to the theory.

By a *cell* we mean a set of *faces* such that each *bar* is a member of two and only two polygonal faces. While we may envisage a cell to be some three-dimensional region of the four-dimensional space, we define it via the skeleton of faces/loops and the bars that comprise them.

By a 4-*polytope* then, we mean a set of *cells* such that every *face* is the member of two and only two cells.

**Figure 2.**
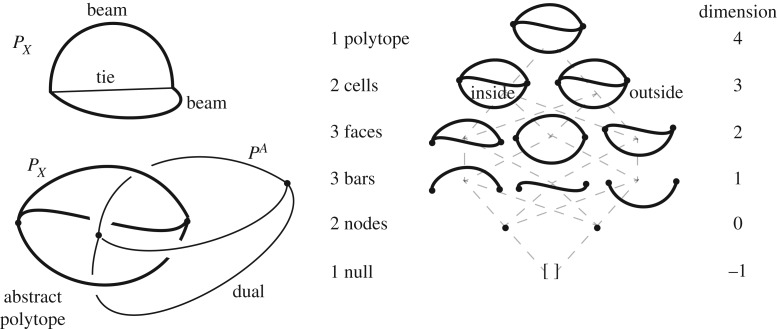
The representation of a simple structure and its stress resultants as dual abstract polytopes. The constituent elements of the structural polytope *P*_*X*_ are arranged in the form of a Hasse diagram (right), the up-down symmetry of which shows the polytope to be topologically self-dual. However, the dual here is not sufficiently rich to obtain meaningful states of self-stress and it will be necessary to add more cells (face cushions) to the original, thereby adding reciprocal nodes along the reciprocal bars (see Example 2 later).

The construction then begins with the definition of a pair of topologically dual *abstract* 4-*polytopes* (partially ordered sets), with duality defined by reading the Hasse diagrams in opposite order. An example is given in figure 2. This first step is purely topological.

Geometry is then introduced by ascribing coordinates in a four-dimensional Euclidean vector space to the nodes and the points on the bars for each polytope *P*_*X*_ and *P*^*A*^.

Note how this already differs substantially from earlier formulations of graphic statics. Traditionally, one is given the geometry of one diagram—the form diagram, say—and one then needs to compute an admissible reciprocal diagram, obeying particular structures such as faces in one diagram being normal to bars in the other. In the new description, given a geometry for *P*_*X*_, then ANY geometry for the topological dual, *P*^*A*^, will correspond to an equilibrium solution. Thus although the abstract nature of the new description may appear to be more complicated, it is in practice considerably easier to implement than the traditional approaches, because no computation is required to compute a reciprocal: any geometry for *P*^*A*^ is admissible.

In a fully general description, the bars of both *P*_*X*_ and *P*^*A*^ may be curved. However, for the present we restrict attention to the case where the bars of the structure *P*_*X*_ may be curved but the bars of the dual polytope *P*^*A*^ must be straight lines between nodes.

Although it would be standard to place points of *P*_*X*_ in a vector space with bases **e**_*i*_, and of *P*^*A*^ in its dual space with bases **e**^*i*^, for present purposes we have no need for index up, index down notation of the basis vectors, and may consider both polytopes to be in the same four-dimensional space, spanned by orthonormal basis vectors **e**_0_,**e**_1_,**e**_2_,**e**_3_ with a standard Euclidean metric.

### 3.2. Structural interpretation: the structure and the stress function

Let the 4-polytope *P*_*X*_ be our structure. Since we presently have no use for the fourth dimension here, we choose to set *x*_0_=1 for all nodes and all points on all bars of *P*_*X*_. That is, our three-dimensional frame lies in the three-dimensional subspace *x*_0_=1 of our four-dimensional setting.

The dual polytope *P*^*A*^ represents the stress function, in that each node **A**^*K*^ of *P*^*A*^ defines the linear stress function over the original cell *C*_*K*_ of *P*_*X*_. That is, the value of the stress function at any point **X** in the cell *C*_*K*_ is given by

3.1 $${\text{A}}^{K}.\text{X}=a^{0}x_{0}+a^{1}x_{1}+a^{2}x_{2}+a^{3}x_{3}.$$

Since we have chosen *x*_0_ to be unity, this is the familiar statement of a linear function over the original structural cell *C*_*K*_ of *P*_*X*_.

To reiterate, a given original cell *C*_*K*_ of the structure *P*_*X*_ has a single dual node **A**^*K*^ in the dual polytope *P*^*A*^. At any point **X** in the original cell *C*_*K*_, the stress function value there is given by the dot product **X**.**A**^*K*^.

Throughout the paper, the phrase ‘stress function’ has two usages. On the one hand, the vertices **A** of the polytope *P*^*A*^ define the set of stress functions which are each linear over their dual cells in the original. In this meaning, the stress function is an abstract object, represented by the polytope *P*^*A*^. This differs from the above description, where the dot product **X**.**A** may be referred to as the ‘stress function’. This, however, is the scalar returned when the stress function is evaluated at point **X**. We shall use both interpretations.

Finally, all interest here focuses on equilibrium states of internal self-stress, and the structures thus carry no external loads. To represent a structure carrying external loads (which is the purpose of most structures), we may—as in previous theories of graphic statics (e.g. [10,11])—partition the structural polytope *P*_*X*_ into a part representing the actual structure and a part representing the external loading. That is, the concept of states of self-stress includes the possibility of representing external loads.

### 3.3. The Corsican sum

Let *P*_*X*_ and *P*^*A*^ be topologically dual 4-polytopes with nodal coordinate vectors {**X**_*J*_=(*x*_0_,*x*_1_,*x*_2_,*x*_3_)=(*x*_0_,**x**)} and {**A**^*K*^=(*a*^0^,*a*^1^,*a*^2^,*a*^3^)=(*a*^0^,**a**)}, respectively. A node *J* of *P*_*X*_ is thus dual to a cell *C*^*J*^ of *P*^*A*^, and a cell *C*_*K*_ of *P*_*X*_ is dual to a node *K* of *P*^*A*^.

Construct the polytope sum *P*_*X*_+_*c*_*P*^*A*^ with nodal coordinates (**X**_*J*_.**A**^*K*^,**x**_*J*_+**a**^*K*^). That is,

3.2 $$P_{X}{+}_{c}P^{A}=\underset{J}{⋃}\;\underset{K\in K(J)}{⋃}{\text{S}}_{J}^{K},\;\mathrm{where}\;{\text{S}}_{J}^{K}=({\text{X}}_{J}.{\text{A}}^{K},{\text{x}}_{J}+{\text{a}}^{K}),$$

where *K*(*J*) is the set of all nodes of the cell *C*^*J*^ of *P*^*A*^ which is dual to the node *J* of *P*_*X*_. For no important reason, we call this a Corsican sum, denoted +_*c*_.

Put simply, each original node **X**_*J*_ in *P*_*X*_ has a dual cell *C*^*J*^ in *P*^*A*^. This dual cell *C*^*J*^ has a number of nodes, *N*(*J*) say, which we may index with *K*, where *K*∈*K*(*J*)={1,…*N*(*J*)}.

We then take the three-dimensional vector coordinates **x**_*J*_ of the node **X**_*J*_, and add the three-dimensional vector coordinates **a**^*K*^ of the *N*_*J*_ nodes of the dual cell *C*^*J*^. That is, we essentially draw the dual cell at each original vertex, with the effect that each original node splits into *N*(*J*) nodes. We then raise each of these new nodes into the fourth dimension by the amount **X**_*J*_.**A**^*K*^. This defines the four-dimensional coordinates of all vertices of the new object, the Corsican sum.

Note that exactly the same set of vertices would have been obtained by drawing, at each node **A**_*K*_ of *P*^*A*^, the original cell *C*_*K*_ of *P*_*X*_ to which it is dual.

The vertices of the Corsican sum are then connected by bars. The topological connectivity is simple to determine. Each original node has split into *N*(*J*) nodes, which are connected with the connectivity of the dual cell *C*^*J*^. Similarly, each dual node *A*^*K*^ has split into *M*(*K*) nodes which are connected with the connectivity of the original cell *C*_*K*_.

**Figure 3.**
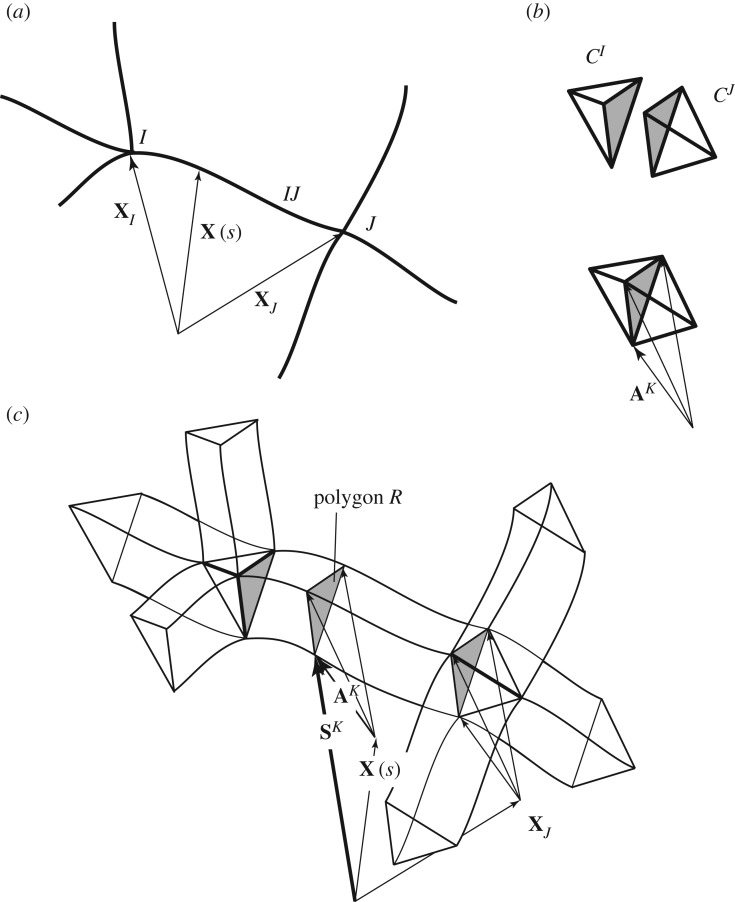
(*a*) A structural bar *IJ* connecting nodes *I* and *J*. (*b*) Cells dual to those nodes. (*c*) Projection of the Corsican sum onto the three-dimensional subspace of **e**_1_, **e**_2_ and **e**_3_. Each point on each line is raised by **X**.**A** in the fourth dimension **e**_0_, but this is not shown.

The construction is illustrated in figure 3. A bar *IJ* connecting nodes *I* and *J* is shown in figure 3*a*. Polyhedra *C*^*I*^ and *C*^*J*^ which are dual to these nodes are shown in figure 3*b*, and as the nodes are connected by a bar, the dual polyhedra share a face, shown shaded. Vector addition of nodal coordinates with the coordinates of the vertices of the appropriate dual polyhedra lead to the Corsican sum, shown in figure 3*c*. Also shown is a point with coordinates **X**(*s*) on bar *IJ*. Vector addition of the coordinates of the point and the vertices of the dual face defines a loop (also shown shaded) in the Corsican sum. It is the various projections of this loop that will define the stress resultant at the point **X**(*s*). Figure 3 is schematic, and somewhat misleading. Strictly the construction occurs in four dimensions, but the behaviour in the fourth dimension is difficult to illustrate. The three-dimensional projection (to **e**_1_,**e**_2_,**e**_3_) of the full construction is exactly as drawn, such that the shaded face is essentially extruded along the bar *IJ*. That this face does not change along the bar is a reflection of the fact that the force vectors (axial plus two shear) do not change along the bar. However, the behaviour in the **e**_0_ direction is given by the dot product **X**.**A** and this has not been shown. This quantity may vary along the bar, reflecting the fact that moments may vary along the bar. In the full four-dimensional construction, then, the ‘thickened’ bars of the Corsican sum are not prismatic (although their three-dimensional projections are).

For present purposes, we restrict attention to the cases where the bars in *P*^*A*^ are straight (otherwise the wedge formula that follows would need to become some more complicated integral). As we have extended the theory, we may also extend the word ‘reciprocal’ to encompass this new duality. We may thus say that the polygon *R* is reciprocal to the bar *IJ* at the point **X**(*s*).

From this construction, we infer that

— reciprocal to a point **X**(*s*) on the bar *IJ* connecting nodes **X**_*I*_ and **X**_*J*_ is a polygon with vertices **S**^*K*^=**X**(*s*)+_*c*_**A**^*K*^, where **A**^*K*^ is any node of *P*^*A*^ which is reciprocal to any cell of *P*_*X*_ of which *IJ* is a common edge.
  The oriented area of this polygon is
  3.3 $$\text{R}(\text{X}(s))=\frac{1}{2}\sum_{K}{\text{S}}^{K}\wedge {\text{S}}^{K+1}\equiv \text{F}+{\text{e}}_{0}\text{M},$$
  where *K*∈*K*(**X**(*s*)) counts over all cells *C*_*K*_ of *P*_*X*_ of which *IJ* is a common edge, and *K*+1 denotes the cell *C*_*K*+1_ adjacent to cell *C*_*K*_ in a clockwise direction when looking onto the cut end of the bar *IJ*, cut at **X**(*s*). The counting is cyclical, such that if the bar is the common edge of *N* cells, then for *K*=*N* we have *K*+1=1. The object **R** is the stress resultant at **X**, containing the force **F** as a bivector and the moment **M** as a vector.

We note in passing the similarity of the stress resultant **F**+**e**_0_**M** of this description with the six-component electromagnetic bivector **E**+*I***B** that is central to GA formulations of electromagnetism [34,35].

### 3.4. Discussion

In previous two- and three-dimensional descriptions, such as Williams & McRobie [12], the reciprocal diagram has been a representation of the forces alone, with the moments manifesting themselves as discontinuities in the stress function over the *form* diagram. In the new four-dimensional description, there is no discontinuity, in the sense that the stress function is completely encapsulated by the reciprocal diagram, which is the dual polytope *P*^*A*^, a continuous, connected object in four dimensions. It only appears to be discontinuous in its three-dimensional manifestation because the stress function evaluated at a point **X**=(1,**x**) on a bar may take (or, more strictly, tend to) any of the values **A**^*K*^.**X** where **A**^*K*^ is the stress function over any of the cells *C*_*K*_ of which the bar is a common edge. That is, earlier descriptions have considered objects of the form (**A**.**X**,**x**), thereby portraying the stress function **A**.**X** as a discontinuous function over the three-dimensional object with nodes {**x**}, with forces represented by a separate three-dimensional object with nodes {**a**}. The new four-dimensional description (**A**.**X**,**a**+**x**) is considerably more coherent and symmetrical.

The four-dimensional object containing items of the form (**A**.**X**,**a**+**x**) is a refinement of the notions proposed in McRobie [33] of Maxwell–Minkowksi and Rankine–Minkowski diagrams for two- and three-dimensional trusses, respectively, where each combined the original and reciprocal diagrams to create a larger object containing both the form and the forces. This new object similarly combines both, but is a continuous four-dimensional object which includes all six components of the stress resultant. Although McRobie [33] referred to such combinations as ‘Minkowski sums’, this does not strictly apply to the new construction, and we thus drop the term ‘Minkowski sum’ in favour of the ‘Corsican sum’, as defined by equation (3.2).

There are a number of ways to generalize the Corsican sum. One such would have terms of the form (*γ***X**.**A**,*α***x**+*β***a**). This merely makes explicit the scale factor between length and force objects that is already implicit in the fundamental definition. By setting *γ*=0 and *β*=1−*α*, and then varying *α* we obtain a continuous sequence of three-dimensional diagrams similar to the three-dimensional Rankine–Minkowski sequences of McRobie [33] which transition between the original and reciprocal structures. Given that four-dimensional information is difficult to display graphically, a number of potential visualization methods—including the use of colour—are described in the examples that follow.

Setting *γ*=1, *α*=1 and *β*=0, we obtain terms of the form (**X**.**A**,**x**). This corresponds to previous methods of formulating the problem, representing the stress function as a discontinuous function over the original three-dimensional structure.

We may similarly obtain terms of the form (**X**.**A**,**a**), this object being the generalization of the Rankine reciprocal. The difference is that it is a four-dimensional object consisting of polygonal faces, these being one-dimensional loops in four dimensions which need not be orthogonal to the bars to which they are reciprocal, and which may be gauche polygons. Again, this is a function over an object with discontinuities at bars. We have restricted attention to the case where the bars in *P*^*A*^ are straight (such that we may use the wedge sum formula). It follows that if we wish to evaluate the stress resultant at a single point, it is irrelevant whether we use **x**+**a** or **a** in the vector slots. The advantage of using the **x**+**a** form only appears when we wish to consider the stress resultants at a number of points, such as on the bar ends exposed by a section cut through the whole structure. In that case, the **x**+**a** keeps the various polygons nicely separated for easier visualization. McRobie [33] and McRobie & Williams [13] discussed the challenges this appears to pose for dimensional analysis: we appear to be adding an object **x** with dimensions of Length to an object **a** with dimensions of $\sqrt{\mathrm{Force}}$. However, as with Rankine–Minkowski diagrams [33], this is not a problem, and all items of physical interest emerge with the correct units.

The scaling we choose for the vector part of the Corsican sum is of far less importance than the fact that we must use **X**.**A** terms in the **e**_0_ slot, rather than some simpler possibility such as *x*_0_+*a*^0^. It is use of the **X**.**A** term that allows us to correctly represent moment equilibrium. Adopting *α*=0, *β*=1, we have terms of the form (**X**.**A**,**a**). If **X** is allowed to move along a bar, there is no change in the **e**_1_, **e**_2_ and **e**_3_ coordinates of the reciprocal polygon vertices. The force in the bar (given by the oriented area in these three dimensions) is thus constant. The moments, however, depend on the **e**_0_ behaviour and thus the moments may vary along the bar. Using **X**.**A** in the first slot ensures that for any two points **x**_1_ and **x**_2_ on a bar, this variation satisfies the moment equilibrium equation **M**(**x**_2_)=**M**_1_(**x**_1_)+(**x**_2_−**x**_1_)×**f**, where **f** is the force (with all terms expressed as vectors in three dimensions). McRobie & Williams [14] present the underlying algebra in full, and we summarize it only briefly here. Since *x*_0_=1 then (**X**.**A**,**a**)=**A**+**D**, with **D**=(**a**.**x**,**0**). Then (**A**+**D**)^*i*^∧(**A**+**D**)^*i*+1^=**A**^*i*^∧**A**^*i*+1^+**D**^*i*^∧**A**^*i*+1^+**A**^*i*^∧**D**^*i*+1^. When summed cyclically over *i* and halved, the first term gives the constant force and constant bending moment. The second and third terms become **e**_0_(**a**^*i*+1^(**x**.**a**^*i*^)−**a**^*i*^(**x**.**a**^*i*+1^)) which, by the vector triple product identity, equals −**e**_0_(**x**×(**a**^*i*^×**a**^*i*+1^)). When summed cyclically over *i* and halved, this gives the differential moment between two points separated by **x** on a bar as −**e**_0_(**x**×**f**), as required. That is, the Corsican sum ensures moment equilibrium via the **X**.**A** term.

There will be a generalization in later sections when we introduce the notion of ‘face cushions’. These are cells possessing only a single polygonal loop, and will be an important element of how the theory endeavours to overcome issues of incompleteness that have hampered previous progress with the Rankine construction.

Finally, the use of **x**+**a** in the Corsican sum appears to lead to various generalizations of Maxwell’s Load Path Theorem. This was the case for the Maxwell–Minkowski diagrams (for two-dimensional trusses, two-dimensional frames and two-dimensional grillages) and Rankine–Minkowski diagrams (for three-dimensional trusses). The essential idea was that the two-dimensional polygons or three-dimensional polyhedra provided a double cover of a region of two- or three-dimensional space. By connecting the original and reciprocal cells with parallelograms/prisms, it followed that the area/volume of the parallelograms/prisms must be equal on each layer of the double cover, and from this we inferred a variety of load path theorems. We do not pursue these ideas in this paper. Instead we now demonstrate how the overall geometric picture may be applied to a number of examples.

## 4. Example 1: the triangular bicycle wheel or 5-cell

A triangular bicycle wheel with six spokes (three either side) connecting to the ends of a central hub is a three-dimensional structural manifestation of one of the simplest 4-polytopes, the 5-cell. Consisting of five conjoined tetrahedra, it provides the higher dimensional analogue of fig. 1 of Maxwell’s 1864 paper [2], which is the projection of the simplest polyhedron, a tetrahedron. The 5-cell has five nodes (which may be labelled 1,2,…), 10 bars (which may be labelled 12,23,…), 10 triangular faces (labelled 123,234,…) and five tetrahedra (1234,2345,…). Its topological dual is another 5-cell, whose nodes may be labelled *A*,*B*,…, bars *AB*,*BC*,…, etc. The duality is readily annotated via complementary labelling, by making node 1 reciprocal to cell *BCDE*, bar 12 reciprocal to face *CDE*, face 123 reciprocal to bar *DE*, etc.

Geometry is specified by picking the four-dimensional coordinates of the five *P*_*X*_ nodes 1,2,… and of the five *P*^*A*^ nodes *A*,*B*,…. Since the duality is topological, these coordinates of both can be chosen freely. (Note how this differs from earlier approaches to three-dimensional graphic statics where, given a structure (a form diagram) the analyst then goes to some trouble to find the geometry of an admissible force diagram. In the new procedure, if *P*^*A*^ is the topological dual of *P*_*X*_, then *any* geometry of *P*^*A*^ is admissible.)

**Figure 4.**
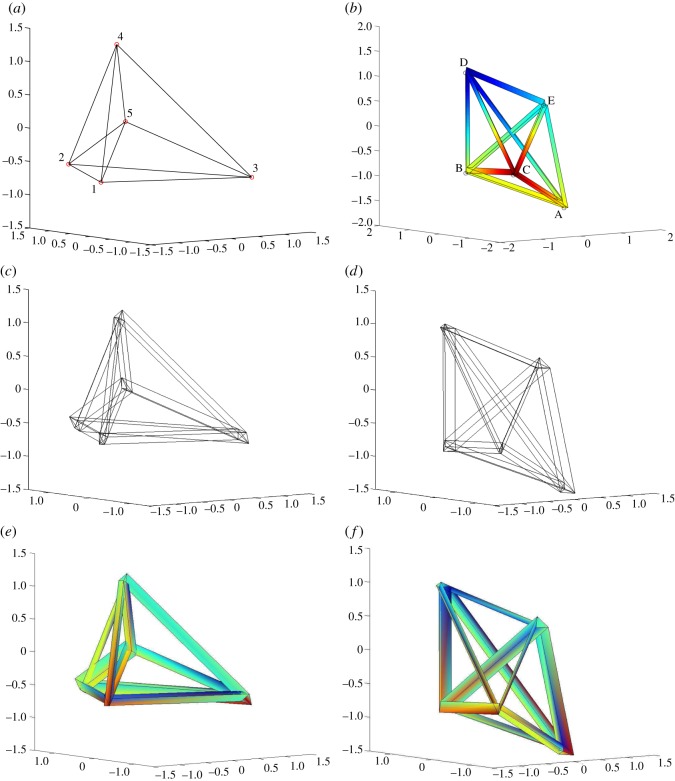
Dual 5-cells. (*a*) The structure (all at *x*_0_=1). (*b*) The dual structure. Values of *a*^0^ in the **e**_0_ direction are indicated by colouring the (artificially thickened) bars. (*c*,*d*) The three-dimensional projection of the Corsican sum for *α*=0.92 and *α*=0.08. Copies of reciprocal cells are evident at original nodes, and vice versa. (*e*,*f*) As above, but with the stress function values **X**.**A** indicated by colouring.

Figure 4*a*,*b* shows a structure *P*_*X*_ and with a dual *P*^*A*^ (albeit that in this configuration, the structure *P*_*X*_ does not look like a triangular wheel). The structure *P*_*X*_ has *x*_0_=1 everywhere, and thus is essentially a three-dimensional object. The dual polytope *P*^*A*^, however, is fully four-dimensional and as ever, there is difficulty in plotting a four-dimensional object on a two-dimensional image. In the visualization shown in figure 4*b*, the reciprocal bars have been artificially thickened and coloured to indicate the coordinate values in the **e**_0_ direction. There is no artificial thickening in the subfigures that follow, the apparent thickening of the bars being a natural consequence of the Corsican sum. Figure 4*c*–*f* shows the Corsican sum at values of *α*=0.92 and 0.08. The skeletal diagram (*c*) illustrates how original nodes are embellished by their reciprocal cells, and (*d*) shows the dual case. In figure 4*e*,*f*, some faces are coloured to indicate the stress function values **X**.**A** in the **e**_0_ direction.

Figure 4*e* is perhaps the most important of the subfigures. The high value of *α*=0.92 means that the diagram resembles the original structure, with bars thickened to represent the forces they carry. However, since reciprocal polygons are no longer orthogonal to bars, we have lost the Lower Bound Plasticity interpretation that was possible for Rankine–Minkowski diagrams. A bar carrying a large shear has a reciprocal polygon inclined relative to the bar. The projection of this polygon perpendicular to the bar direction can indeed represent the amount of material necessary to carry the axial forces, but additional material would be needed to carry the shear forces (and the moments).

The representation of the stress function components associated with the moments by means of colouring is also far from ideal. While it leads to an interesting graphic, it is difficult to interpret visually, and later examples will look at alternative graphical procedures for conveying the moment information. However, it nevertheless illustrates some important points. First, it reminds us that the Corsican sum contains all the information about the stress resultants. Second, it can be seen that the continuity of the colouring reflects how the Corsican sum is a continuous object. Third, the fact that colours vary around any bar cross-section reflects how approaching the *α*=1 representation (the stress function over the line-like structure) leads to a stress function which is discontinuous on bars.

Despite the difficulties of creating an intuitive visual presentation, all stress resultant information can nevertheless be extracted algebraically to obtain the equilibrium state of stress in the original structure *P*_*X*_ that is defined by this choice of reciprocal structure *P*^*A*^. Finally, we note that not all possible equilibrium states of self-stress of this structure can be captured by its representation via dual 5-cells. It was shown by McRobie & Williams [14] that to be able to represent any stress resultant in a bar, the bar would need to be the common edge of at least five cells. In the 5-cell, however, each bar is common to only three cells, and one consequence is that at any point the forces will be necessarily orthogonal to moments (when each is represented by a three-dimensional vector). This limitation can be readily overcome by the introduction of ‘face cushion’ cells, but we leave that for a later section.

## 5. Example 2: tied arches

**Figure 5.**
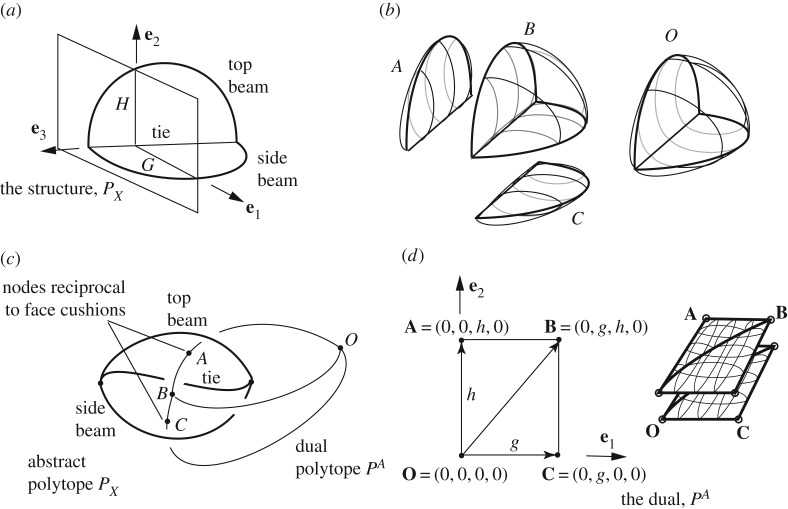
(*a*) The structure *P*_*X*_ consisting of two curved beams and a tie. (*b*,*c*) Abstract polytope representation. Cushions have been added to horizontal and vertical faces of the structure. (*d*) The dual polytope *P*^*A*^.

Figure 5*a* shows a simple structure *P*_*X*_. It consists of two curved beams with a tie. Given that much of the literature on polytopes concerns regular polytopes with plane faces and straight edges (such as the tesseract) it may not be immediately apparent that the simple structure has a dual polytope representation. However, the abstract polytope of figure 2 earlier corresponds to exactly this case. The dual polytope there, though, is not sufficiently rich to represent any interesting states of self-stress. However, given the generality of the formulation here, this problem can be readily overcome by simply adding more cells to the original. These cells have a form that we choose to call ‘face cushions’. These are cells which have only two surfaces, each spanning the same single polygonal loop of bars. That is, they resemble a cushion padding the face in question. A face cushion is a cell, thus adding a face cushion means introducing an additional reciprocal node along the reciprocal bar that was dual to the original face. This freedom to add face cushions (and corresponding reciprocal nodes) almost anywhere is key in providing the richness of the solution space which thereby enables a wide set of equilibrium states to be represented by the new description. It is even permissible (and sometimes necessary) to add more than one face cushion to a face.

In the tied beam example here, we choose to add two face cushions, one each on the horizontal and vertical faces, such that the original, *P*_*X*_, now has four cells (figure 5*b*).

The abstract polytope representation is shown in figure 5*c*. In this new approach, it is no longer necessary to calculate an admissible reciprocal. Rather, we are now free to choose any geometry for the dual polytope *P*^*A*^. For simplicity of explanation, we choose the simple arrangement shown in figure 5*d*.

**Figure 6.**
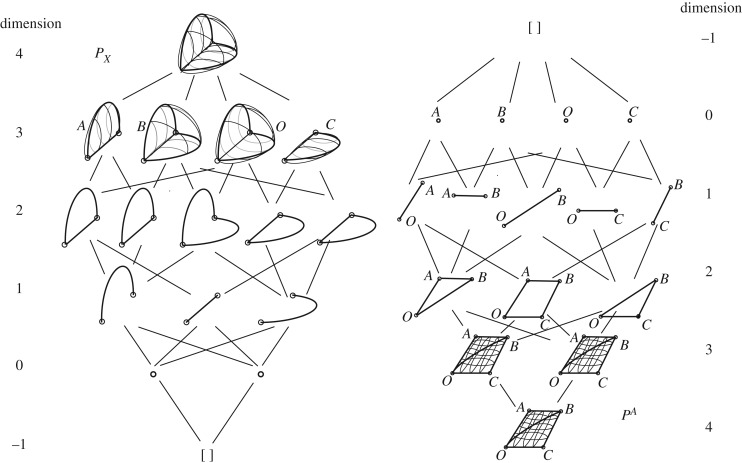
Hasse diagrams of the structure *P*_*X*_ and its topological dual *P*^*A*^. The structure (with the inclusion of the face cushions) has four cells, thus the dual has four nodes. The structure has two nodes such that the dual has two cells.

By way of further clarification, the Hasse diagrams showing the topological duality of the polytopes *P*_*X*_ and *P*^*A*^ are shown in figure 6. With the inclusion of the face cushions, the structure *P*_*X*_ has four cells, thus the dual has four nodes. Similarly, the structure has two nodes such that the dual has two cells. From a geometrical perspective, these two cells may be considered to have zero thickness, since all face polygons lie within the **e**_1_, **e**_2_ plane. Nevertheless, the generality of the previous definitions admits the possibility of such three-face polyhedra of arbitrary or even zero thickness, and the two polyhedra together provide the double cover necessary for the definition of the polytope *P*^*A*^.

**Figure 7.**
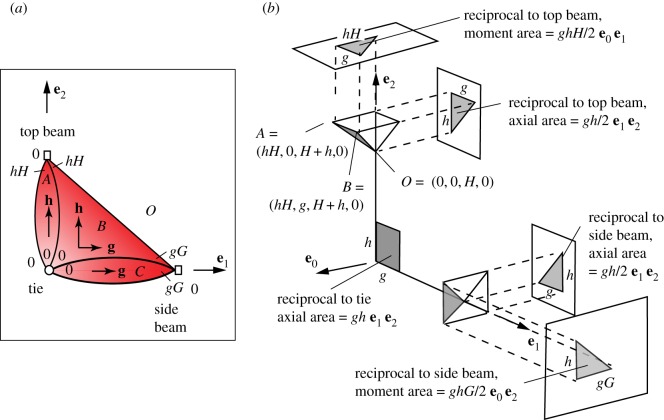
(*a*) A transverse section through the four cells *A*,*B*,*C*,*O* of *P*_*X*_ at constant *z*. (*b*) A section at constant *z* through the Corsican sum, with projected areas giving the forces and bending moments.

Since *P*_*X*_ and *P*^*A*^ are now defined, topologically and geometrically, the Corsican sum can now be constructed. A section at constant *z* is shown in figure 7*a*. Since **e**_3_ information is not required, we use the third dimension to display **e**_0_ information. Reciprocal to the points on each bar is a polygon whose oriented area defines the stress resultant. For this choice of *P*^*A*^, the tie carries an axial force of *gh* which is resisted equally by the two beams. Since all forces are given by **e**_1_**e**_2_ bivectors (i.e. corresponding to a vector in the **e**_3_ direction), these are not orthogonal to the beams, which thus carry axial and shear forces. The bending moments are given by the **e**_0_**e**_1_ and **e**_0_**e**_2_ bivector components of the reciprocal polygons. These have magnitudes *ghH*/2 and *ghG*/2, and are in moment equilibrium with the system of internal forces.

The example demonstrates that a graphical method exists for determining the equilibrium stress resultants in a rather general three-dimensional frame. Given the history of graphic statics, with its focus on polyhedral structures with straight bars, and with an emphasis on constructing a ‘valid’ reciprocal, it is striking that the new description so readily allows the analysis of a structure (figure 5*a*) with curved bars that seems extremely distant from all previous theory of graphic statics.

Many further complications may be introduced into this example, and the general procedure still works. For example, the beams could be very general three-dimensional space curves, even though it may not then be immediately apparent that the structure has any representation as a polytope. Nevertheless, the representation as dual abstract polytopes would be identical to that here. It was shown in McRobie & Williams [14] that to generate all possible stress resultants in a beam, that beam must be the common edge of at least five cells and the reciprocal face must be gauche. In the example here, the curved beams are the common edge of only three cells, but additional face cushions can be readily introduced to create a dual polytope that is rich enough to represent all possible states of self-stress.

## 6. Example 3: Rankine incompleteness for three-dimensional Trusses

The overall framework that has been described in this paper is a natural generalization of Rankine’s reciprocal construction for three-dimensional trusses to the case of three-dimensional frames. Having developed such a general method for three-dimensional frames, we now apply it back to some earlier problems that have plagued the Rankine construction for three-dimensional trusses. For this, we do not even need the full four-dimensional methodology, but can work purely in three dimensions.

Maxwell [3] identified that Rankine’s description is incomplete in that there can exist three-dimensional truss states of stress which have no Rankine reciprocal. The new procedure described in this paper appears capable of avoiding any such limitations, and while we do not yet have a full proof of completeness either for frames or for trusses, we give a few examples of how some archetypal problematic configurations may now be readily dealt with. Throughout, the key features of the new description that appear to surmount previous problems are the ability to deal with gauche polygons, and the freedom to add as many face cushions as desired.

**Figure 8.**
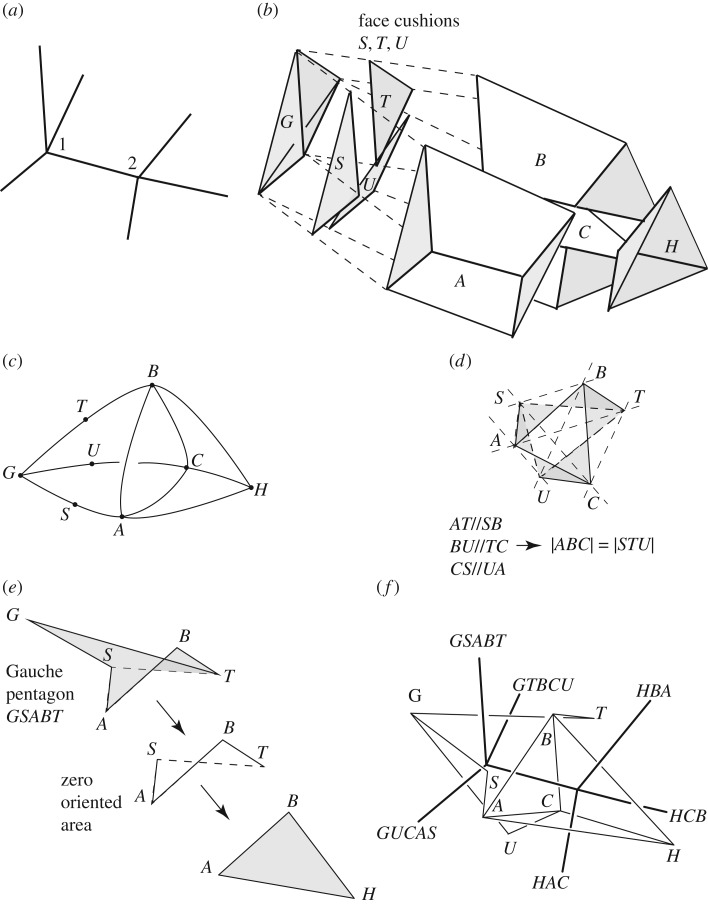
(*a*) If the seven bars shown are in general position, then this part of a truss has no Rankine reciprocal. (*b*) Face cushions *S*, *T* and *U* may be introduced to isolate the left-hand tetrahedron *G*. (*c*) Additional reciprocal nodes are thus introduced into the topology, creating three pentagonal faces. (*d*) The new reciprocal nodes may be placed in the plane *ABC* in such a way as to introduce no additional oriented area. (*e*) The gauche pentagon *GSABT* decomposes to a triangle and a Zero Bar quad. (*f*) The oriented areas of the seven faces are perpendicular to the seven original bars.

A standard problem for Rankine reciprocals concerns a bar that has 4-valent nodes at each end (figure 8*a*). For each node, there is a reciprocal tetrahedron but they cannot be conjoined to assemble an overall Rankine diagram: although the two triangles reciprocal to the connecting bar have the same area, their geometry may differ.

The configuration is a three-dimensional variant of the classic two-dimensional Desargues arrangement wherein two triangles are connected by three bars. In that case, there is a two-dimensional truss state of self-stress if and only if the lines of the three connecting bars meet at a point. The three-dimensional generalization here has tetrahedra *G* and *H* connected by four bars. If the lines of these four bars meet at a point, then a three-dimensional truss state of self-stress can be readily modelled with a Rankine reciprocal. However, there also exist truss states of self-stress where the four connecting lines do not meet at a point. The configuration then contains a number of gauche quad polygons. To encapsulate the problem for the purposes of illustration, we restrict attention to the inner seven truss members, assuming there is some wider outer frame capable of resisting any resulting forces. As drawn, we have seven bars in the problematic morphology where no three of the seven are coplanar.

Initially, there are five cells of interest, the end tetrahedra *G* and *H* and three prism-like polyhedra *A*, *B* and *C* that connect them. The incompleteness problem is solved by adding face cushions *S*, *T* and *U* over the faces of the tetrahedron *G* to separate it from the prism-like cells *A*, *B* and *C* (figure 8*b*). The topological connectivity of the cells is shown in figure 8*c*. Without face cushions, the topological dual has topological tetrahedra with a common face *ABC* (the topological viewpoint ignoring the possibility that the faces may not match geometrically). The addition of the face cushions results in there being additional reciprocal nodes *S*, *T* and *U* on the bars of the left-hand (topological) tetrahedron, which then possesses one triangular and three pentagonal faces.

We now introduce geometry. First we create the two basic Rankine tetrahedra *GSTU* and *HABC* as usual for each nodes 1 and 2, each independent of the presence of the other. Triangles *STU* and *ABC* are coplanar and of equal area, but differ geometrically. From any initial placement, the left tetrahedron *GSTU* can be moved in an arbitrary direction until lines *AT* and *SB* are parallel, and then moved along the direction parallel to *AT* until *BU* and *TC* are parallel. (Moving an object corresponds to applying an additional constant gradient stress function over its original cells.) It then follows that quads *ABTS* and *BCUT* each have zero oriented area. As triangles *STU* and *ABC* have equal area, it follows that quad *CASU* has zero oriented area. The resulting geometry on the two-dimensional plane orthogonal to bar 12 is shown in figure 8*d*.

Each pentagonal face can be expressed as the sum of a triangle and a quad, as per figure 8*e*, where the triangle *GST* is that of the original Rankine, and the quad is a Zero Bar. The force in that member is the oriented area of the pentagon, which thus equals the original Rankine tension |*GST*| along the bar, plus zero. Collecting all the elements together creates two conjoined polyhedra whose polygonal faces have oriented areas orthogonal to the bars. As each is a closed polyhedron, we thus have zero total oriented area, this corresponding to a state of equilibrium where each bar carries an axial force equal to the area of the reciprocal polygon. The result is shown in figure 8*f*, and this is a generalized Rankine diagram for the two problem nodes.

## 7. Example 4: the irregular octahedron

**Figure 9.**
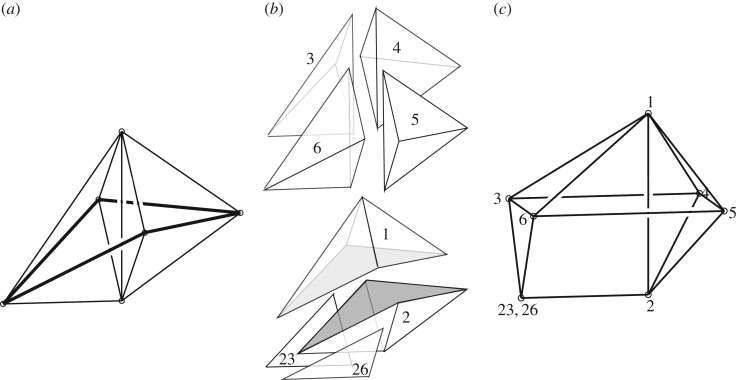
(*a*) An irregular octahedron with central spindle. (*b*) The decomposition into cells, including two face cushions. (*c*) The generalized Rankine reciprocal for the purely axial state of self-stress.

This is another example of a three-dimensional truss, an octahedron with a central spindle, as was considered by McRobie [36]. The structure resembled a square, horizontal bicycle wheel with two sets of spokes connecting to the ends of a central vertical hub. The problem was that if the wheel was warped then any face that spanned it was gauche (figure 9*a*), and the Rankine reciprocal for the purely axial state of self-stress could not be constructed. By adding a Zero Bar across a diagonal of the gauche square to divide it into two triangles, all cells were then tetrahedral and a Rankine reciprocal could be constructed having no force in the Zero Bar. Here, an alternative strategy is presented. Instead of adding a Zero Bar, face cushions are added over two of the triangular faces (figure 9*b*). The wheel is still a warped square, but gauche polygons are no problem for the new theory and a generalized Rankine reciprocal is readily constructed (figure 9*c*). Because the topology of the structure differs from that of McRobie [36] (no Zero Bar, two face cushions), the reciprocal structures also differ, even though the purely axial state of self-stress is the same for both.

## 8. Example 5: gridshell rooves

From the three-dimensional truss examples, we return to the analysis of moments in three-dimensional frames. Here we suggest a practical two-stage strategy for modelling real—and potentially rather complicated—structures such as gridshell rooves. The first stage looks only at the forces, and can be solved in a three-dimensional setting in a simple and highly visual manner. The second stage addresses the moments, and must be done in four dimensions. However, as the example will demonstrate, even there the method provides substantial visual insight.

### 8.1. Force equilibrium in three dimensions

The first part of the strategy is via the somewhat remarkable geometric fact that by simply dividing a three-dimensional frame into constituent polyhedra and then translating each one, the gaps between the nodes of the shifted polyhedra define an equilibrium set of forces.

We consider some simple roof examples, but before even considering the roof itself we consider the force system that exists below the roof and which represents the roof loads and the supporting columns. We take examples where the roof nodes are laid out on a rectilinear grid, with each internal roof node loaded by a vertical wire and with the four corner nodes supported by vertical columns.

**Figure 10.**
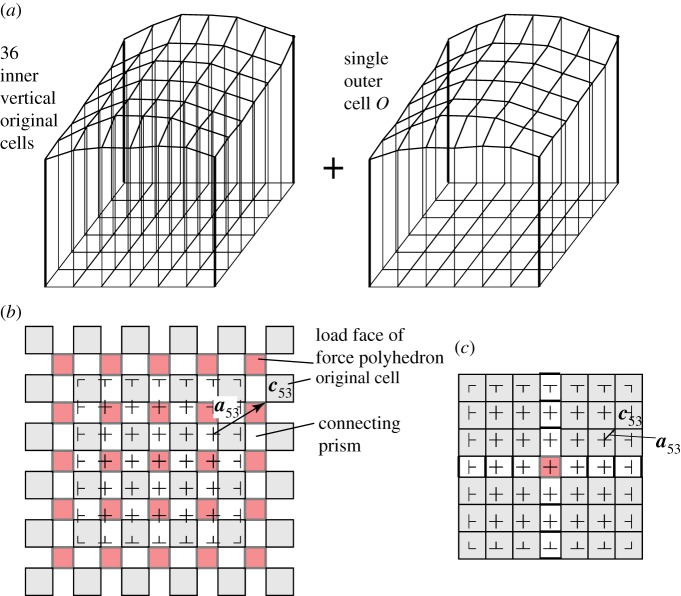
Loads on a gridshell roof. (*a*) The polyhedral double cover, consisting of a set of vertical prisms with skew ends, all contained within a single outer polyhedron with many faces. The horizontal cross sections of (*b*,*c*) show horizontal translations of the prism-like original cells that create, respectively, equal loads on each inner roof node, and a concentrated load at the central node only. The forces are given by the areas of the red shaded polygons that now exist transverse to every original bar.

Figure 10*a* shows a loaded roof, and the covering polyhedra consist of a set of vertical prisms with skew ends together with a single surrounding polyhedron. Two load cases are shown, with figure 10*b* applying equal vertical loads to each internal roof node and figure 10*c* applying a single point load at the centre. In each case, all polyhedra have been translated in the *x*,*y*-planes, thus all gaps are planar polygons orthogonal to the vertical lines of force, the oriented areas of these polygons giving the forces. More general loadings can be created in this manner by simply translating the polyhedra in different directions in three dimensions. The act of translating the polyhedra corresponds to building the Corsican sum, with each translation being the gradient **a** of the stress function across that cell, or equivalently, the three-dimensional coordinates **a** of the reciprocal node of the dual polytope.

The reciprocal polygons created by this method exist at present only within three dimensions, but they need not be orthogonal to the bar direction and they may be gauche, although—for simplicity—not in the examples given here.

**Figure 11.**
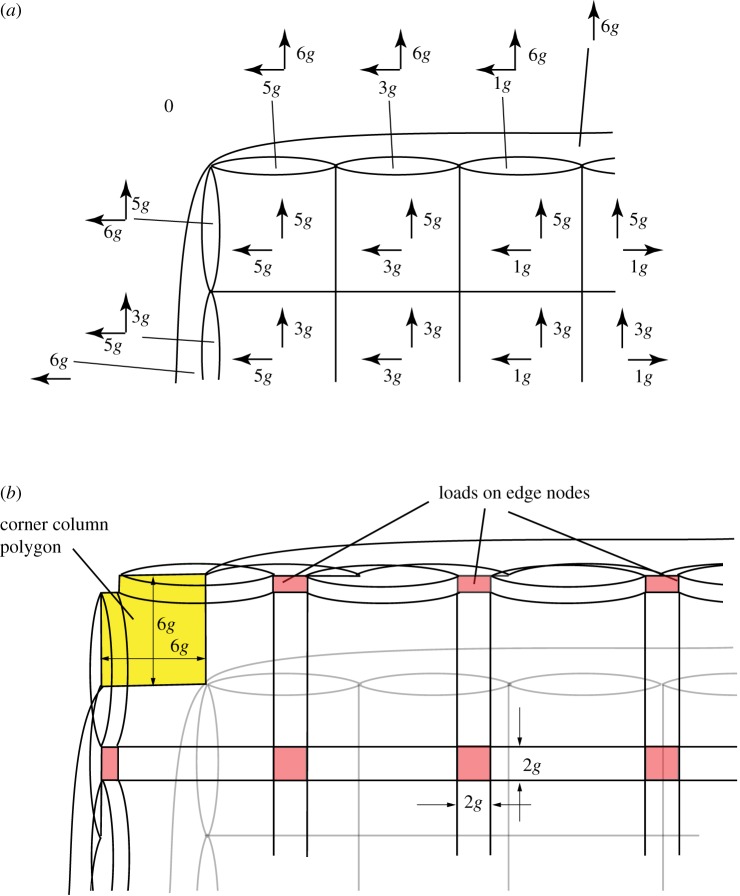
(*a*) One quadrant of a horizontal section beneath a square roof with 36 roof panels. Face cushions are inserted into each wall panel and a larger cushion is applied over each wall. (*b*) Appropriate translation of the cells creates polygons reciprocal to the equal loads on each inner roof node, and half-loads on each edge node. All load is resisted by the corner columns. From this second diagram, the appropriate stress function gradients can be written on the original cells back on the first diagram (*a*).

In standard graphic analysis, gridshells are typically modelled as funicular three-dimensional pin-jointed trusses, not as three-dimensional moment-bearing frames, as here. A difficulty can then arise at the edges, where thrusts from the roof arch action reach the roof edge. The funicular shells then need to adopt inclined shapes at the roof edges. However, for a rectilinear gridshell such as shown in figure 10, no horizontal thrust can be carried at the edges by truss action alone: the edge beams must carry moments, which can now be readily modelled within the new description using face cushions. Let us say that the edge nodes have no columns beneath them, but are loaded by vertical wires like the inner roof nodes. Say these wires at the edge nodes apply half the load applied at an internal node. There are many ways that face cushions can be defined to recreate this case, but since there is great freedom, we opt for a neat solution. The strategy is to place reciprocal nodes almost wherever one wants in order to create a neat diagram having the correct polygon areas, and then take the coordinates of these reciprocal nodes as the stress function gradients over the face cushions. Figure 11*a* shows just such an arrangement. The inner roof nodes are loaded by squares of area 4*g*^2^ such that it would be neat to have the roof edge nodes loaded by rectangles of area 2*g*×*g* laid out as shown. Appropriate face cushions on each wall panel readily create this pattern and additional outer face cushions over each whole wall help to keep the diagram simple. In the example shown there are 25 inner wires applying 4*g*^2^ each, and 20 edge wires each applying 2*g*^2^. The total load of 140*g*^2^ is then resisted by the four corner columns which each carry (6×6−1=35)*g*^2^.

Clearly, the method is far more general than in the example shown here, but it is hoped that it can nevertheless be seen that this first part of the procedure is rather simple: spread out the vertical prism cells to create the desired loading pattern and then translate face cushions in wall panels to create the desired edge conditions.

### 8.2. Moment equilibrium (four-dimensional)

In the three-dimensional procedure above, each vertical line of force now has a reciprocal polygon representing the axial force, and appropriate choices for the *a*^0^ stress function values can ensure that these vertical members carry no coexistent moments. For this, the full four-dimensional procedure is required.

**Figure 12.**
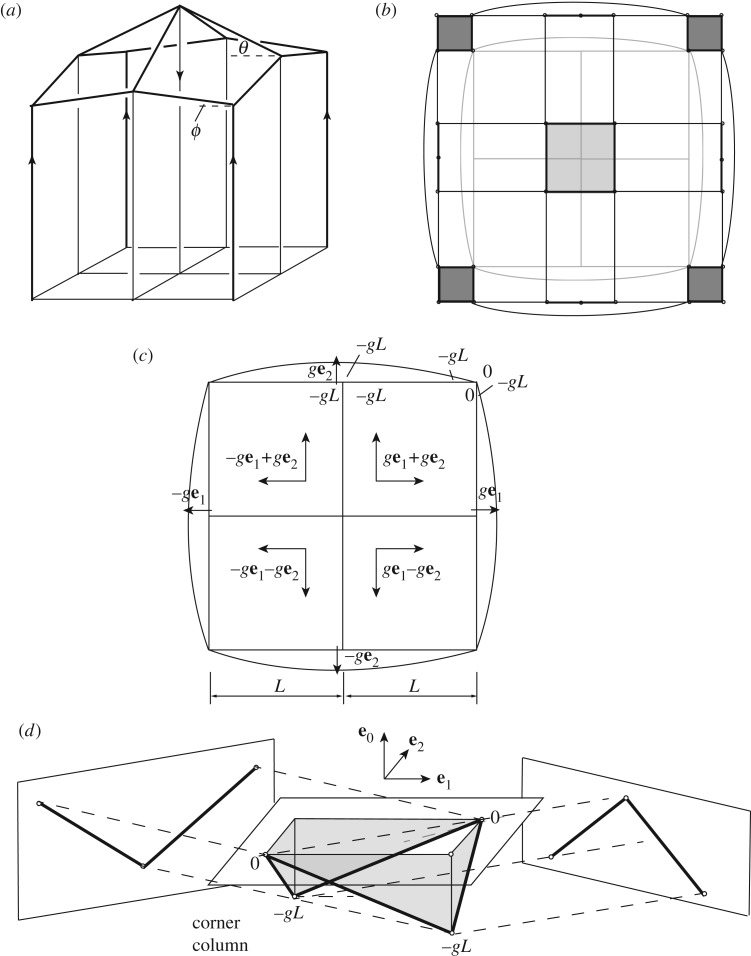
(*a*) A simple gridshell roof. (*b*) A horizontal section showing cell translations that lead to zero force in the midwall verticals. (*c*) The resulting stress function gradients. (*d*) The polygon reciprocal to the corner columns has zero projected area on the **e**_0_(**e**_1_+**e**_2_) and **e**_0_(**e**_1_−**e**_2_) planes.

To simplify the example for clarity, consider a roof with four roof panels (figure 12*a*) supporting only a central point load which is carried ultimately by four corner columns. The roof panels can be gauche polygons. Again, before looking at the roof members we consider the loading system by taking a horizontal cross-section below the roof. The three-dimensional method of the previous section can readily ensure that midwall verticals carry no axial load. However, we also require that these carry no moment.

The three-dimensional procedure for forces has defined the gradients **a** of the stress function for each polyhedron. These are shown in figure 12, with (*b*) showing the translations, and (*c*) showing these expressed as stress function gradients **a** over each cell. The four-dimensional procedure now focuses on providing the *a*^0^ constant term for each cell in a manner that will create the desired loading arrangement. A suitable set of choices are shown on figure 12*c*. There is no discontinuity around the midwall vertical, thus there is no moment. The polygon reciprocal to the corner column is shown in figure 12*d*, where we have suppressed the **e**_3_ direction (which is irrelevant), allowing display of the **e**_0_ information associated with the moments. Two orthogonal projections are shown, from which it is clear that there is zero oriented area on both planes, thus the corner column carries no moment. These results can be readily derived using the wedge formula applied to the values shown in figure 12*c*, but since we are developing a graphical method, it is of more interest to see the results displayed graphically.

### 8.3. The roof members

The stress function has now been defined over every cell below the roof, and there exists a corresponding state of stress in the roof members. At any point on any roof member, the stress resultants can be computed either algebraically or graphically for this stress function. However, a designer has freedom to select other states of roof stress by adding face cushions into the roof panels. Different choices of the face cushion stress functions then lead to different states of stress in the roof. We present one possible solution—one in which the designer wishes the roof cross-beams to carry purely axial forces.

**Figure 13.**
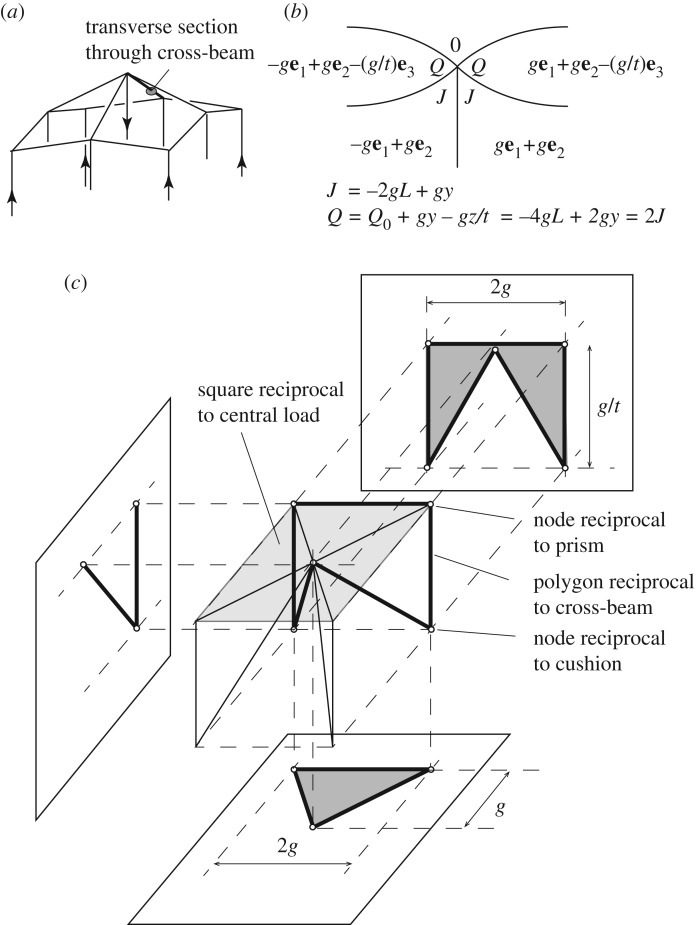
(*a*) The cross-beam section of interest. (*b*) The cell connectivity on the section transverse to the cross-beam. (*c*) The pentagon reciprocal to the cross-beam, and the projections of its oriented area. These are *g*^2^ vertically and *g*/*t* horizontally such that the overall oriented area is orthogonal to the beam.

Figure 13*a* shows the cell connectivity on a section transverse to a roof cross-beam, with face cushions added to the roof panels. Since the roof beam is then the common edge of five cells, its reciprocal is a topological pentagon. Three of its nodes are defined, but the designer is free to choose the coordinates **A** for the two nodes reciprocal to the face cushions. The suitable choice is most readily seen by looking at the roof apex, where the four cross-beams meet the applied central load. The polygon for the central load is a horizontal square, and each cross-beam will carry a triangular quarter of this. Indeed, the three known nodes for the cross-beam coincide with the corners of this triangle. Horizontal force can be generated by lowering the two nodes reciprocal to the roof cushions. We place them below the corners of the square, and lower them sufficiently to generate a horizontal force such that the total force is aligned along the cross-beam. Elementary geometry shows that lowering them by −*g*/*t***e**_3_ (where t=tan⁡θ, the cross-beam inclination) gives the desired result. That is, the resulting reciprocal pentagon has an oriented area of *g*^2^ vertically and *g*/*t* horizontally in the beam direction, such that the resultant force is aligned along the beam.

Back at the apex, the pentagons for the four cross-beams can be connected around the square reciprocal to the central load to create a closed polyhedral cell reciprocal to the upper node. This is a cuboid but with its underside hollowed out by a pyramid-shaped hole (figure 13*c*). It is a closed polyhedron, and we thus have nodal force equilibrium.

We choose the (*x*,*y*,*z*) coordinate origin to be at the roof apex, and we wish to determine the stress function constants *q*_0_ for the roof cushions that give zero moment in the cross-beams. The appropriate value is *q*_0_=−4*gL*. The point reciprocal to the right-hand face cushion (figure 13*b*) is thus **Q**=(−4*gL*,*g*,*g*,−*g*/*t*). Adjacent to the bar, at point **X**=(1,0,*y*,−*ty*), the stress function value is thus **X**.**Q**=*Q*=−4*gL*+0+*gy*−(*g*/*t*)(−*ty*)=−4*gL*+2*gy*=2*J*, where *J* is the stress function in the vertical prism below. Substituting this into the wedge formula confirms that there is then no moment in the cross-beam.

**Figure 14.**
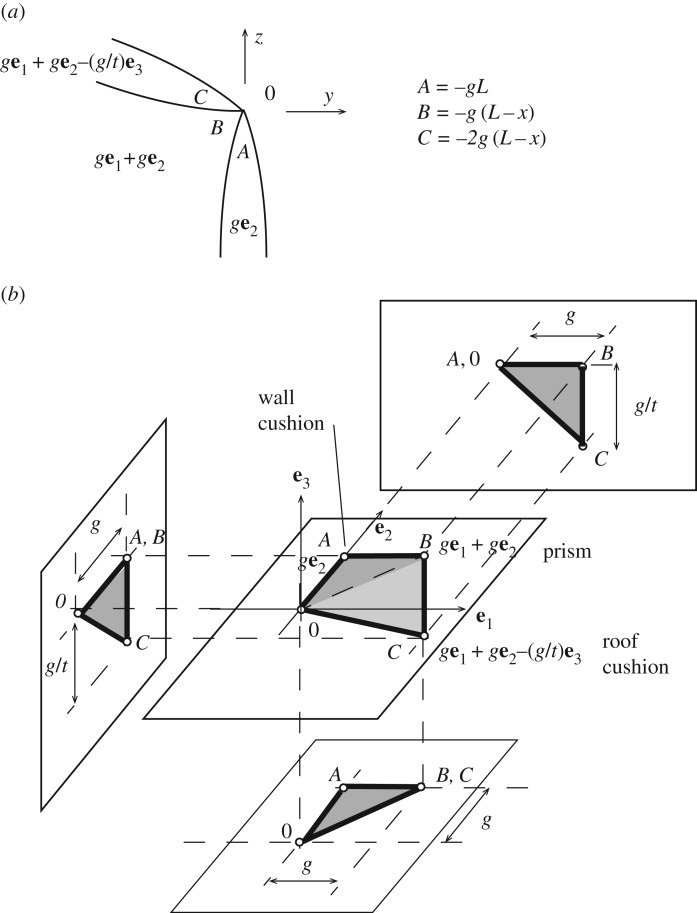
(*a*) The cell connectivity on a section transverse to an edge beam. (*b*) The gauche quadrilateral reciprocal to the edge beam (in three dimensions, with **e**_0_ information omitted), and the projections of its oriented area that give the forces.

All stress function values have now been determined, and it remains only to evaluate the stress resultant in the edge beams. Although the procedure works even if the roof beam inclinations *θ* and *ϕ* are different (and the roof panels are thus gauche), we proceed for brevity with the case *θ*=*ϕ*. The cell connectivity on a section transverse to an edge beam is shown in figure 14*a*. To calculate the forces we plot and connect the gradients of the four cells in three dimensions, temporarily suppressing the **e**_0_ information. This gives a gauche quad (figure 14*b*) whose projections give the force components.

**Figure 15.**
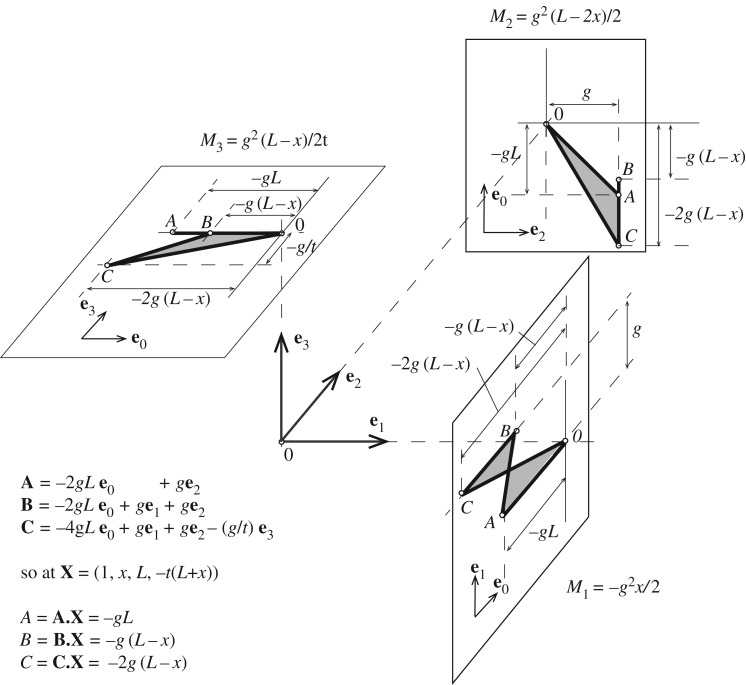
Edge beam moments displayed as oriented areas. The points of the Corsican sum are plotted on the **e**_0_**e**_*i*_(*i*=1,2,3) subspaces. These are placed such that the oriented area is orthogonal to the **e**_*i*_ direction corresponding to the moment vector *M*_*i*_.

To calculate the moments, we need the *a*^0^ terms for each cell in order to determine the stress function values adjacent to any point on the beam. These have all been determined in the preceding analysis, and are shown in the legend of figure 15. In particular, at the point (1,*x*,*L*,−*t*(*L*+*x*)) on the beam, we have the roof cushion stress function value *C*=−4*gL*+*gx*+*gL*+(−*g*/*t*)(−*t*(*L*+*x*))=−2*g*(*L*−*x*). The figure also demonstrates one possible method for illustrating the moment information. The moments are given by the **e**_0_**e**_*i*_ bivector areas, but the four-dimensional nature of the problem creates visualization difficulties. Here the bivector areas **e**_0_**e**_*i*_ have been presented on the **e**_*j*_**e**_*k*_ planes (*i*≠*j*≠*k*), such that they appear as areas oriented perpendicular to the vector that would usually represent them. The final diagram is not particularly instructive. Nevertheless, it is evident that, whether algebraically or graphically, the moments can be determined. More intuitive graphical methods for displaying them may yet be devised, failing which one can always simply plot the moments via traditional methods.

In this case, the edge beams carry the horizontal component *g*^2^/*t* of the thrust from the cross-beams in a simply supported manner, with the moment *M*_3_=−*g*^2^(*L*−*x*)/2*t* varying from the standard *WL*/4 value of (*g*^2^/*t*)(2*L*)/4=*g*^2^*L*/2*t* at the centre (*x*=0) to zero at the support (*x*=*L*). The vertical component of the applied load is carried in a combination of arch action and bending. In particular, the torsion is $M_{1}\cos\theta -M_{3}\sin\theta =-(g^{2}L\cos\theta )/2$, which is a constant, as it must be in the absence of applied torques. This torsion equilibrates with the end moment in the orthogonal edge-beam to which it connects at the corner, in the sense that the *M*_1_ and *M*_2_ moments of one beam at the corner equilibrate with the corresponding *M*_2_ and *M*_1_ components of the orthogonal edge beam.

The designer has thus found a set of stress resultants in equilibrium with the applied loads, and which have purely axial forces in the cross-beams as desired. The behaviour of the edge beams is considerably more complicated, each carrying axial and shear forces with coexistent torsion and bending moments. Nevertheless, all details can be distilled from the graphical construction. The solution here is not complete, in that other states of stress can exist in the edge beams which are in equilibrium with the applied loads and roof thrusts. However, while these could be readily mobilized by incorporating additional face cushions, we do not pursue that here for sake of brevity.

Finally, we note that there is a grillage at the base of the construction which is loaded by the central and corner forces. There is an equilibrium state of stress in this grillage which can readily be computed. However, there will often be little need to do so. The grillage can find internal forces that self-equilibrate with any loads that are applied to it. The procedure here thus obviates the need to determine the geometric details of any funicular-like system below the roof such as is sometimes used to create the applied loading on the roof above.

In summary, the two-stage procedure of applying forces in a three-dimensional setting and moments in a four-dimensional setting is both practical and powerful, and is arguably simpler to apply in practice than the formalism of the underlying mathematics may at first suggest. Moreover, the freedom to add face cushions at will makes the method extremely general.

## 9. Summary and conclusion

The paper has presented a generalization of Rankine’s reciprocal construction for three-dimensional trusses to the case of three-dimensional frames. The key was the definition of dual abstract 4-polytopes, whose vertices were points in dual four-dimensional vector spaces which were described using four-dimensional Clifford algebra. Rankine’s notion of a force being represented by a polygonal area orthogonal to the bar was generalized to having a stress resultant (with all six components of axial, shear, torsion and bending) represented by the oriented area of a general polygon in four dimensions. These polygons do not need to be orthogonal to the bar, can be gauche and may have general curvilinear edges. This new description not only allows access to the analysis and design of rather general three-dimensional frames, but also resolves a number of long-standing problems in Rankine’s description of three-dimensional trusses. Finally, examples were given of how to apply the theory in practice, including an outline of a two-stage procedure for tackling real structures such as gridshell rooves.

A purely geometric description of the equilibrium of structural frames has been presented, and the similarity of the six-component stress resultant bivector **F**+**e**_0_**M** to the electromagnetic bivector **E**+*I***B** is something that would presumably have interested Maxwell who, between 1860 and 1870, wrote the definitive papers on both subjects, each founded on duality and geometry.
