# Correction to ‘The geometry of structural equilibrium’

**DOI:** 10.1098/rsos.170338

**Published:** 2017-05-10

**Authors:** Allan McRobie

*R. Soc. open sci.* **4**, 160759 (Published online 22 March 2017). (doi:10.1098/rsos.160759)

## 1. Introduction

This correction concerns fig. 9 of 1. The diagram illustrates how the state of self-stress in an irregular octahedron with a central spindle can be found by introducing ‘face cushion’ cells onto the octahedral surface. In the original publication, the face cushions are incorrectly placed in fig. 9*b* and the resulting Rankine reciprocal is incorrect in fig. 9*c*. The correct arrangements are shown below.

**Figure 9.**
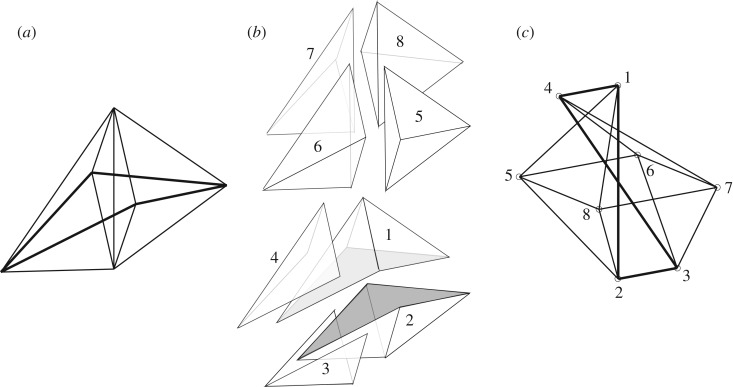
(*a*) An irregular octahedron with central spindle. (*b*) The decomposition into cells, including two face cushions. (*c*) The generalized Rankine reciprocal for the purely axial state of self-stress.

## References

[1] McRobie A 2017 The geometry of structural equilibrium. R. Soc. open sci. 4, 160759 (doi:10.1098/rsos.160759) 28405361 10.1098/rsos.160759 PMC5383818

